# A Strange Freak

**Published:** 1876-01

**Authors:** 


*A Strange Freak.*—The *New York Herald* states that Miss Sarah Ward, aged 28, daughter of Judge Ward, who resides at Tompkinsville, Staten Island, visited a New York dentist on Monday last to have some teeth extracted, and took laughing gas. She remained under the influence of the gas for a considerable time, and when she finally recovered the idea seemed to have struck her that it would be a good joke to frighten her folks at home by telegraphing to the Rev. A. N. Stanley, rector of St. Paul's, that she was dead. She accordingly sent a dispatch to the rector, who was preaching at the time in observance of St. Andrew's day, that she died from the effects of inhaling laughing gas. The startling announcement caused great consternation among the congregation, the young lady being well known to them all. The services were at once concluded, and word was sent to her father, who hastened to the dentist's place of business, where he was surprised as well as overjoyed to learn that his daughter had but a short time previously left for home in excellent health. When asked by her parents what induced her to send such a dispatch, she said that she did it for fun.

